# FIG: The Finite Improbability Generator

**DOI:** 10.1007/978-3-030-45190-5_27

**Published:** 2020-03-13

**Authors:** Carlos E. Budde

**Affiliations:** 8 Johannes Kepler University, Linz, Austria; 9 University of Birmingham, Birmingham, UK; Formal Methods and Tools, University of Twente, Enschede, the Netherlands

## Abstract

This paper introduces the statistical model checker FIGV, that estimates transient and steady-state reachability properties in stochastic automata. This software tool specialises in Rare Event Simulation via importance splitting, and implements the algorithms RESTART and Fixed Effort. FIG is push-button automatic since the user need not define an importance function: this function is derived from the model specification plus the property query. The tool operates with Input/Output Stochastic Automata with Urgency, aka IOSA models, described either in the native syntax or in the JANI exchange format. The theory backing FIG has demonstrated good efficiency, comparable to optimal importance splitting implemented ad hoc for specific models. Written in C++, FIG can outperform other state-of-the-art tools for Rare Event Simulation.

## 1 Introduction

In formal analysis of stochastic systems, statistical model checking (SMC [33]) emerges as an alternative to numerical techniques such as (exhaustive) probabilistic model checking. Its partial, on-demand state exploration offers a memory-lightweight option to exhaustive explorations. At its core, SMC integrates Monte Carlo simulation with formal models, where traces of states are generated dynamically e.g. via discrete event simulation. Such traces are samples of the states where a (possibly non-Markovian) stochastic model usually ferrets. Given a temporal logic property $\varphi$ that characterises certain states, an SMC analysis yields an estimate $\hat{\gamma}$ of the actual probability $\gamma$ with which the model satisfies $\varphi$. The estimate $\hat{\gamma}$ typically comes together with a quantification of the statistical error: two numbers $\delta \in (0,1)$ and $\varepsilon > 0$ such that $\hat{\gamma} \in [\gamma - \varepsilon, \gamma + \varepsilon]$ with probability $\delta$. Thus, if $n$ traces are sampled, the full SMC outcome is the tuple $(n, \hat{\gamma}, \delta, \varepsilon)$.

With this statistical quantification—usually presented as a confidence interval (CI) around $\hat{\gamma}$—an idea of the quality of an estimation is conveyed. To increase the quality one must increase the *precision* (smaller $\varepsilon$) or the *confidence* (bigger $\delta$). For fixed confidence, this means a narrower CI around $\hat{\gamma}$. The number of traces $n$ is inversely proportional to $\varepsilon$ and to the CI width, so SMC trades memory for runtime or precision when compared to exhaustive methods [5].

This trade-off of SMC comes with one up and one down. The **up** is the capability to analyse systems whose stochastic transitions can have non-Markovian

✠ This work was partially funded by NWO, NS, and ProRail project 15474 (*SEQUOIA*) and EU project 102112 (*SUCCESS*).

The Author(s) 2020
A. Biere and D. Parker (Eds.): TACAS 2020, LNCS 12078, pp. 483–491, 2020.
https://doi.org/10.1007/978-3-030-45190-5_27

distributions. In spite of gallant efforts, this is still out of reach for most other model checking approaches, making SMC unique. The **down** are rare events. If there is a very low probability to visit the states characterised by the property $\varphi$, then most traces will not visit them. Thus the estimate $\hat{\gamma}$ is either (an incorrect) 0 or, if a few traces do visit these states, statistical error quantification make $\varepsilon$ skyrocket. To counter such phenomenon, $n$ must increase as $\gamma$ decreases. Unfortunately, for typical estimates such as the sample mean, it takes $n \geqslant {}^{384}/_{\gamma}$ to build a (rather lax!) CI where $\delta = 0.95$ and $\varepsilon = \frac{\gamma}{10}$. If e.g. $\gamma \approx 10^{-8}$ then $n \geqslant 38400000000$ traces are needed, causing trace-sampling times to grow unacceptably long. Rare Event Simulation (RES [24]) methods tackle this issue.

The two main RES methods are importance sampling (IS) and importance splitting (ISPLIT). IS compromises the aforementioned **up**, since it must tamper the stochastic transitions of the model. Given that the study of non-Markovian systems is a chief reason to use SMC, FIG, a statistical model checker specialised in RES, implements ISPLIT. To deploy an efficient implementation, however, both importance sampling and splitting require expert knowledge. The novelty of FIG lies on its automatic derivation of the importance function (and thresholds and splitting values) required by ISPLIT. This derivation exploits the model and property under study, resulting in a push-button application of RES for SMC.

**Outline.** The way in which FIG approaches RES is explained in Sec. 2. Its model and properties input syntax are presented in Sec. 3. Finally, Sec. 4 mentions some features of FIG 1.2, before ending the paper with the briefest experimental display.

**Related work.** Other statistical model checkers offer RES methods to some degree of automation. Plasma Lab implements automatic IS [18] and semiautomatic ISPLIT [21] for Markov chains. Its ISPLIT engine offers a wizard that guides the user to choose an importance function. The wizard exploits a layered decomposition of the property query—not the system model. Via APIs, the ISPLIT engine of Plasma Lab could be extended beyond DTMC models. $\mathcal{S}$BIP 2.0 [22] implements the same (semiautomatic, property-based) engine for DTMCs. $\mathcal{S}$BIP offers a richer set of temporal logics to define the property query in. Cosmos [1] and FTRES [26] implement importance sampling on Markov chains, the latter specialising in systems described as repairable Dynamic Fault Trees (DFTs). All these tools can operate directly on Markovian models, and none offers fully automated ISPLIT. Instead, the SMC tool modes [5] supports non-Markovian probability distributions and is much closer to the capabilities of FIG, offering a similar degree of automation. As a matter of fact, all core RES algorithms in modes were inspired in or motivated by the theory behind FIG. On the one hand, FIG is restricted to fully-stochastic IOSA models, whereas modes can also cope with nondeterminism (e.g. in Markov automata) using the LSS algorithm [10, 5]. On the other hand, using the batch means method, FIG can estimate steady-state properties, which modes cannot currently do. Moreover, FIG 1.2 implements basic functionality to tailor importance functions for DFTs.

Previous versions of FIG have been used for scientific experimentation and research: the theory of [6] was first implemented and exercised with FIG 1.0; and FIG 1.1 was presented in [2], and last used in an extended journal version of [5].

## 2 Rare Event Simulation

RES methods make more traces visit the rare states that satisfy a property $\varphi$ (the set $\mathcal{S}_\varphi$), to reduce the variance of SMC estimators. For a fixed budget of traces $n$, this yields more precise CIs than classical Monte Carlo simulation (CMC).

FIG implements *importance splitting*: a main RES method that can work on non-Markovian systems without special considerations. ISPLIT splits the states of the model into layers that wrap $\mathcal{S}_\varphi$ like an onion. Reaching a state in $\mathcal{S}_\varphi$ from the surface is then broken down into many steps. The $i$-th step estimates the conditional probability to reach (the inner) layer $i+1$ from (the outer) layer $i$. This stepwise estimation of conditional probabilities can be much more efficient than trying to go in one leap from the surface of the onion to its core [20].

Formally, let $\mathcal{S}$ be the states of a model with initial states $\mathcal{S}_0$ and rare states $\mathcal{S}_\varphi$. ISPLIT works on a partition $\biguplus_{i=0}^{M} \mathcal{S}_i = \mathcal{S}$, where $\mathcal{S}_\varphi = \mathcal{S}_M$. To estimate the probability $\gamma = \mathit{Prob}(\mathcal{S}_\varphi \,|\, \mathcal{S}_0)$, each conditional probability $\gamma_i = \mathit{Prob}(\mathcal{S}_i \,|\, \mathcal{S}_{i-1})$ is estimated separately via CMC. Then simply $\hat{\gamma} = \prod_{i=1}^{M} \hat{\gamma}_i \approx \prod_{i=1}^{M} \gamma_i = \gamma$.

This approach is correct, i.e. it yields an unbiased estimator $\hat{\gamma} \xrightarrow{n\to\infty} \gamma$. However, it is efficient iff $\forall_{i=1}^{M} . \gamma_i \gg \gamma$, which depends on how the $\mathcal{S}_i$ layers where chosen. For this, an *importance function* $f\colon \mathcal{S} \to \mathbb{R}_{\geqslant 0}$ and *thresholds* $\ell_i \in \mathbb{R}_{\geqslant 0}$ are defined: then $\mathcal{S}_i = \{s \in \mathcal{S} \mid \ell_i \leqslant f(s) < \ell_{i+1}\}$, where $\ell_0 = 0$, and $\mathcal{S}_\varphi$ are the states with highest *importance*, i.e. $f(s) \geqslant \ell_M$. The efficiency of ISPLIT is thus delegated to the choice of $\{\ell_i\}_{i=1}^{M}$ and the importance function $f$.

These choices are *the* key challenge in ISPLIT [20]. Theoretical developments assume $f$ is given [12, 8], and applications define it ad hoc via (RES and domain) expert knowledge [30, 27]. Yet there is one general rule: importance must be proportional to the probability of reaching $\mathcal{S}_\varphi$. Thus for $s, s' \in \mathcal{S}$, if a trace that visits $s'$ is more likely to observe a rare state, one wants $f(s) \leqslant f(s')$. This means that $f$ depends both on the model $\mathsf{M}$ and the property $\varphi$ that define $\mathcal{S}_\varphi$.

FIG, an SMC tool, exploits the formal definitions of $\mathsf{M}$ and $\varphi$ to derive $f$ and $\{\ell_i\}_{i=1}^{M}$ so as to reflect this rule. For this, FIG runs BFS from $\mathcal{S}_\varphi$ on the (inverted) transitions of $\mathsf{M}$. This computes the number-of-transitions distance from each state to $\mathcal{S}_\varphi$. The heuristic importance function of FIG, $f^\star$, is the inverse of this distance, stored as an array the size of $\mathcal{S}$. To avoid the state explosion FIG works on modular formalisms, deriving local $f_i^\star$ for the $\mathsf{M}_i$ whose parallel composition forms $\mathsf{M}$. $f^\star$ is an aggregation of these functions, e.g. adding the $f_i^\star$ of every $\mathsf{M}_i$ with variables in $\varphi$. Details are in [2] and also in [5], where the difference with the (later) implementation in modes is that FIG uses the DNF of $\varphi$.

$f^\star$ is solely based on the number-of-transitions distance. Stochastic behaviour of $\mathsf{M}$ omitted by $f^\star$, such as probabilistic labels in the transitions, is captured in the thresholds $\ell_i$. For this, FIG runs short simulations that start from $\mathcal{S}_0$. Say $K_1$ out of $N$ simulations visit states with importance $i_1 > i_0 = f^\star(\mathcal{S}_0)$. Then, 1 out of $e_1 = \left\lceil \frac{N}{K_1} \right\rceil$ simulations are expected to reach threshold $\ell_1 = i_1$. Next, repeat this procedure starting from states with importance $i_1$ to choose $\ell_2$ and $e_2$. Etc. Such threshold-selection algorithms (see Sec. 4) are fully described in [4].

Thus, just from $\mathsf{M}$ and $\varphi$, FIG enables ISPLIT by computing $f^\star$ and $\{\ell_i, e_i\}_{i=1}^{M}$.

## 3 Modelling formalism and input languages

**IOSA.** FIG models are Input/Output Stochastic Automata with urgency [11]. In IOSA, continuous variables called *clocks* sample random values from arbitrary distributions (PDFs). As time evolves, all clocks count down at the same rate. The first to reach zero can trigger events and synchronise with other modules, broadcasting an *output* action that synchronises with homonymous *input* actions (IOSA are input-enabled). Actions can be urgent, where urgent outputs have maximal progress. IOSA can thus be nondeterministic: to allow simulation, [23] gives conditions to ensure determinism modulo weak bisimulation. IOSA *variables* are clocks, integers, or Booleans. *Constants* can also be floats and have global scope (variables are module-local). FIG offers array variables and can get e.g. "a-random/the-smallest value." Code 1 shows the guarded command language of FIG models. Decorators ?/! tell an action is input/output, e.g. fl!. Double decorators (r??) are for urgency. Non-urgent outputs can be sent only on clock expiration ([fl!]···@fc->). A clock can sample random values (fc'=$\mu$).

```
module M1
  fc,rc   : clock;
  inf,brk : [0..2] init 0;
  [fl!] brk==0 @ fc -> (inf'=1)
                    & (brk'=1);
  [r??] brk==1 ->(brk'=2) & (rc'=γ);
  [up!] brk==2 @ rc -> (inf'=2)
                    & (brk'=0)
                    & (fc'=μ);
  [f!!] inf==1 -> (inf'=0);
  [u!!] inf==2 -> (inf'=0);
endmodule
```

Code 1: IOSA module in FIG 1.2

**JANI.** Besides its native input syntax, FIG 1.2 reads models written in the JANI exchange format [7]. Model types supported are CTMC and a subset of STA that matches IOSA, e.g. with a single PDF per clock and broadcast synchronisation. FIG also translates IOSA to JANI as STA, to share models with tools such as the MODEST TOOLSET [16] and Storm [13]. This is used in Sec. 4 for comparisons.

**Properties.** FIG estimates the probability with which input models satisfy temporal logic formulæ. A formula is specified as a (transient or steady-state) property query in the model file. Transient properties in FIG correspond to the PCTL-like query P=? in PRISM [19]: e.g. the first property in Code 2 asks the probability of assigning value 8 to variable q2 before it takes a value $\leqslant 0$. Steady-state properties in FIG correspond to the unbounded CSL-like query S=? in PRISM: e.g. S(q2>=8). For steady-state estimations FIG implements batch means [9]. The initial (discarded) transient simulation time, and the batch time, can be heuristically computed by the tool. These values can also be given by the user—in Code 2, the last property specifies 9 and 999 resp.

```
properties
  P( q2>0 U q2==8 )
  S( q2>=8 )
  S[9:999]( q2>=8 )
endproperties
```

Code 2: Property queries in FIG

## 4 FIG 1.2 showcase

The *Finite Improbability Generator* is written in C++14 and is available at https://git.snt.utwente.nl/buddece/fig under the GNU GPLv3. FIG is built in modules across three categories: simulation engines, importance functions, and thresholds builders. Engines are nosplit, restart, and sfe, which resp. run CMC, RESTART (RST [31]), and Fixed Effort (FE [14]) simulations. The latter two are ISPLIT algorithms: FE was described in Sec. 2, and works for transient properties; RST also works for steady-state analysis (steady-state via FE requires regeneration theory [15], seldom applicable to non-Markovian models and unsupported by FIG 1.2). RST and FE work with an *effort* $e$. $FE_e$ means $e$ simulations are ran in a layer $\mathcal{S}_i$. $RST_e$ means $e-1$ clones are spawned when a simulation up-crosses a threshold $\ell_i$. Omitting $e$ makes FIG 1.2 use respectively $FE_8$ or $RST_3$.

A RES run yields a random value $r \in [0,1]$ of unknown distribution, so FIG computes standard CLT confidence intervals with Student's $t$-distribution quantiles. $r$ has a Bernoulli distribution only for transient properties estimated with CMC: FIG can then use Wilson score intervals [32]. Floating-point precision loss is reduced by using the logarithm of $r$ and of the number of runs.

FIG reads or computes importance functions. Option `--adhoc` takes as mandatory argument a function on the variables of the IOSA modules. Instead, `--amono` automatically builds $f^\star$ on the parallel composition of all modules, and `--acomp` builds a local $f_i^\star$ per IOSA module—see Sec. 2. For `--acomp`, FIG takes an optional argument to aggregate all local $f_i^\star$ into one global $f^\star$. This can be an associative binary arithmetic operator, or a custom function on the names of the IOSA modules. By default, $f^\star$ is computed as the sum of all local functions. Option `--dft 0` indicates that the model is a fault tree: FIG then builds specialised local importance functions for certain modules, e.g. basic events and PAND gates.

Two algorithms in FIG 1.2 can compute the thresholds and efforts $\{\ell_i, e_i\}_{i=1}^{M}$. Sequential Monte Carlo [8, 6] (SEQ, option `-t hyb`) is characterised by one effort for all regions $\mathcal{S}_i$, set with `-g` $e$. Instead, Expected Success [4] (ES, `-t es`) determines each effort $e_i$ per $\mathcal{S}_i$ region. By default FIG 1.2 uses `-e restart -g 3 -t hyb`. Other customisable options are the RNG, its seed, the floating point precision, and a timeout. Mandatory arguments for FIG invocation are the model and properties file, the simulation type (`--flat` for CMC, or `--adhoc/amono/acomp` for RES), and a stop criterion (either time, or confidence and precision of the CI).

**Experimental demonstration.** We display the capabilities of FIG via three experiments. First, we show how ISPLIT implemented in FIG 1.2 is as automatic but more efficient than CMC to estimate rare properties. Second, we test the degree to which $f^\star$ in FIG can approximate optimal importance functions chosen ad hoc for some models. Third, we compare FIG and its closest competitor: modes. All these experiments can be reproduced via the artifact freely available in [3].

We test different configurations of engines, efforts, and thresholds. For each configuration we run simulations until some timeout. This yields a CI with precision $2\varepsilon$ for confidence coefficient $\delta = 0.95$. The smaller the $\varepsilon$, the narrower the CI, and the better the performance of the configuration (and tool) that produced it.

First, we analyse repairable DFTs with warm spares and exponential (fail), normal (repair), and lognormal (dormancy) PDFs. Using CMC, $FE_{8,16,32}$ and $RST_{3,4,6}$ we estimate the probability of a top level event after the first failure, before all components are repaired, in trees with 6, 7, and 8 spares (the smallest IOSA has 116 variables and $> 2.5\,\mathrm{e}\,37$ states). For ISPLIT we used SEQ thresholds with `--dft 0 --acomp` and no arguments, i.e. as automatic as CMC.

With a 20 min timeout, each configuration was repeated 13 times in a Xeon E5-2683v4 CPU running Linux x64 4.4.0. The height of the bars in the top plot of Fig. 1 is the average CI precision (lower is better), using Z-$\text{score}_{m=2}$ to remove

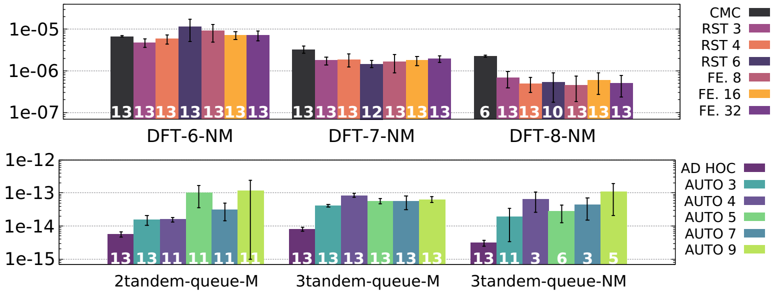

Fig. 1: CI precision. Top: DFTs (transient). Bottom: queues (steady-state).

outliers [17]. Whiskers are standard deviation, and white numbers indicate how many runs yielded not-null estimates. Clearly, RES algorithms outperform CMC in the hardest cases: less than half of CMC runs in DFT-8 could build (wide) CIs.

Second, we estimate the steady-state overflow probability in the last node of tandem queues, on a Markovian case with 2 buffers [29], 3 buffers [28], and a non-Markovian 3-buffers case [30]. We study how FIG—using `--amono`, SEQ, and RST$_{3,4,5,7,9}$—approximates each optimal ad hoc function and thresholds of [29, 28, 30]. Experiments ran as before: the bottom plot of Fig. 1 shows that FIG's default (RST$_3$ with SEQ, legend "AUTO 3") is always closest to the optimal.

Third, we compare FIG and modes in the original benchmark of the latter [5]. We do so for FE-SEQ, RST-SEQ, RST-ES, using each tool's default options. We ran each benchmark instance 15 min, thrice per tool, in an Intel i7-6700 CPU with Linux x64 5.3.1. The scatter plots of Fig. 2 show the median of the CI precisions. Sub-plots on the bottom-right are a zoom-ins in the range $[10^{-10}, 10^{-5}]$.

An (x,y) point is an instance whose median CI width was x for FIG 1.2 and y for modes netcore-3.0.150, single threaded. A point over the solid diagonal line means FIG built a narrower CI. A point on the upper boundary means that modes built no CIs in all runs. Dotted diagonal lines indicate CIs twice as wide. Fig. 2 shows that both tools perform similarly, with a slight trend in favour of FIG. This could be caused by modes operating on JANI STA (translated from IOSA by FIG): modes must assign values to variables and then compare them to clocks.

Albeit modes is multi-threaded, these experiments ran on a single thread to compare both tools on equal conditions. On the other hand, FIG also estimates the probability of steady-state properties, for which there is no support in modes.

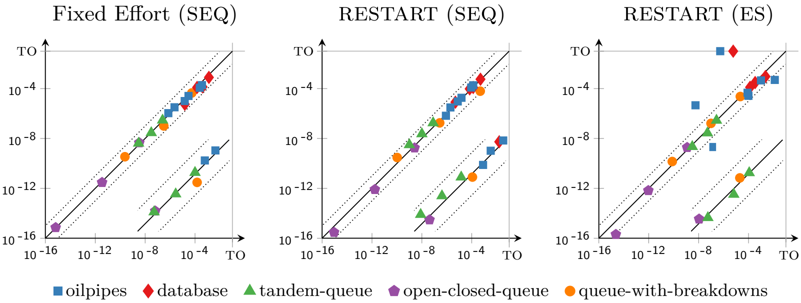

Fig. 2: CI precision of FIG (x-axis) vs. modes (y-axis): medians of 3 runs × 15 min
